# Transcriptomic analysis reveals the key role of histone deacetylation via mediating different phytohormone signalings in fiber initiation of cotton

**DOI:** 10.1186/s13578-022-00840-4

**Published:** 2022-07-12

**Authors:** Zhenzhen Wei, Yonghui Li, Faiza Ali, Ye Wang, Jisheng Liu, Zuoren Yang, Zhi Wang, Yadi Xing, Fuguang Li

**Affiliations:** 1 Zhengzhou Research Base, State Key Laboratory of Cotton Biology, Zhengzhou University, Zhengzhou, 450001 China; 2 State Key Laboratory of Cotton Biology, Institute of Cotton Research, Chinese Academy of Agricultural Sciences, Anyang, 455000 China; 3 National Nanfan Research Institute (Sanya), Chinese Academy of Agricultural Sciences, Sanya, 572024 Hainan China; 4 Sanya Institute, Zhengzhou University, Sanya, 572024 Hainan China

**Keywords:** Fiber initiation, Histone deacetylation, HDAC inhibitor, TSA, Phytohormones

## Abstract

**Background:**

Histone deacetylation is one of the most important epigenetic modifications and plays diverse roles in plant development. However, the detailed functions and mechanisms of histone deacetylation in fiber development of cotton are still unclear. HDAC inhibitors (HDACi) have been commonly used to study the molecular mechanism underlying histone deacetylation or to facilitate disease therapy in humans through hindering the histone deacetylase catalytic activity. Trichostatin A (TSA)—the most widely used HDACi has been extensively employed to determine the role of histone deacetylation on different developmental stages of plants.

**Results:**

Through in vitro culture of ovules, we observed that exogenous application of TSA was able to inhibit the fiber initiation development. Subsequently, we performed a transcriptomic analysis to reveal the underlying mechanisms. The data showed that TSA treatment resulted in 4209 differentially expressed genes, which were mostly enriched in plant hormone signal transduction, phenylpropanoid biosynthesis, photosynthesis, and carbon metabolism pathways. The phytohormone signal transduction pathways harbor the most differentially expressed genes. Deeper studies showed that some genes promoting auxin, Gibberellic Acid (GA) signaling were down-regulated, while some genes facilitating Abscisic Acid (ABA) and inhibiting Jasmonic Acid (JA) signaling were up-regulated after the TSA treatments. Further analysis of plant hormone contents proved that TSA significantly promoted the accumulation of ABA, JA and GA_3_.

**Conclusions:**

Collectively, histone deacetylation can regulate some key genes involved in different phytohormone pathways, and consequently promoting the auxin, GA, and JA signaling, whereas repressing the ABA synthesis and signaling to improve the fiber cell initiation. Moreover, the genes associated with energy metabolism, phenylpropanoid, and glutathione metabolism were also regulated by histone deacetylation. The above results provided novel clues to illuminate the underlying mechanisms of epigenetic modifications as well as related different phytohormones in fiber cell differentiation, which is also very valuable for the molecular breeding of higher quality cotton.

**Supplementary Information:**

The online version contains supplementary material available at 10.1186/s13578-022-00840-4.

## Background

Histone acetylation functions as one type of the most important chromatin modifications in eukaryotes, which generally is mediated by the opposite functions of histone acetyltransferases (HATs) and histone deacetylases (HDACs) to sustain the balance of lysine acetylation for regulation of gene expression in context [1]. Many HDAC genes have been identified and characterized from animals, fungi and plants to involve diverse developmental stages or stress tolerances [2–9]. Up to now, HDACs were classified into three subfamilies in eukaryotes. The first is most closely related to yeast RPD3, and the second is related to yeast HDA1 [10]. Moreover, the third subfamily of HDACs (HD2) is unrelated to yeast RPD3 and is unique in plants [11–14]. HDACs generally function as a part of multiprotein complexes consisting of transcriptional repressors, scaffold proteins, and a variety of cofactors [8].

In mammals and humans, HDACs play vital roles in cell migration, growth, and survival, and are closely correlated with various diseases such as tumors, cancers, a group of diseases related to metabolic abnormalities, and so on [7, 15, 16]. HDAC inhibitors (HDACi) can repress histone deacetylation mediated by HDAC through several different approaches and have been studied widely to be applied in diverse disease therapies. According to the different structures and characteristics of the HDAC subfamilies, a variety of HDACi were identified and designed to cure human diseases [17–20]. Some HDACs function with zinc in their catalytic site, hence the main target of HDAC inhibitors is to suppress the inherent activity of HDACs by occupying the catalytic core of the Zn^2+^ binding site [15, 21, 22]. Trichostatin A (TSA) and vorinostat [suberoylanilide hydroxamic acid (SAHA)] have been identified to inhibit HDACs catalytic activity, among which TSA was the first discovered natural hydroxamate and HDACi used in treating patients with malignancy together successfully with vorinostat (e.g., cutaneous T-cell lymphoma) [7, 23, 24]. Furthermore, some aminosuberoyl hydroxamic acids have shown the ability to repress HDACs and cell proliferation at nanomolar concentrations [21], facilitating the application of HDACi in disease therapy. Basically, HDACi promotes the accumulation of acetylated histones and non-histone proteins that are involved in the regulation of gene expression, enzymatic activity, cell proliferation, and so on. Even so, the molecular mechanisms underlying the HDACi-mediated cell growth retardation and cell death associated with antitumor are complex and not completely elucidated [17, 25–27]. The development of small molecule HDACi for various disease conditions including cancer, is an emerging target in recent times [16].

In insects, life-history traits such as longevity and fecundity are severely affected by the suppression of HAT/HDAC activity, achieved by the application of chemical inhibitors. Bea aphid (*Acyrthosiphon pisum*)-a model insect is often used to study complex life-history traits. Specific chemical inhibitors of HATs/HDACs showed a remarkably severe impact on life-history traits including reducing survival, delaying development, and limiting the number of offspring. The selective inhibition of HATs and HDACs also had opposing effects on aphid body weight [5].

Furthermore, many studies have proved that HDACs play critical and versatile roles in plant development including seed germination, vegetative and reproduction tissues development, trichome development, root hair cell differentiation, as well as abiotic stress tolerance [2, 3, 5, 6, 28]. In *Arabidopsis*, it also showed that TSA and diallyl disulfide (DADS) inhibited the seedling development in MS medium [28]. In rice, using TSA resulted in impaired callus formation of mature embryos and increased global histone H3 acetylation levels, decreased auxin response, and cell proliferation in callus formation [29]. Cotton fiber, the principal natural resource for the textile industry, is also an excellent model to study cell differentiation and development. Thirty HDAC genes were identified from the tetraploid variety *Gossypium hirsutum*, and of them, *GhHDA5* has shown the negative regulation of fiber cell differentiation, indicating some clues for the mechanisms of HDACs and the crucial roles of histone deacetylation in fiber development [6]. However, the underlying mechanism associated with histone deacetylation for fiber development is unclear.

The application of HDACi in plants also provided some interesting results. For example, plant cell cultures are good for the output of recombinant proteins, with lower costs than mammalian cells except the only flaw being the lower yields obtained. After adding HDACi into the culture, higher levels of transgene expression and protein accumulation were observed, showing HDACi as an enhancer of recombinant protein production in plant cell suspensions. This offers the potential to improve the yields of the recombinant protein in plant cell cultures with epigenetic strategies [30], providing evidence of the correlation between histone acetylation and increased transcription levels and the production of recombinant proteins. Moreover, TSA showed negative roles in fiber development through in vitro culture of ovules [6], supporting the key role of histone deacetylation in fiber development.

Here, the HDACi-TSA was used for in vitro culture of ovules before and after fiber cells initiation (i.e. ovules at 0 DPA and −2 DPA). The results revealed that TSA inhibits not only fiber elongation, but also fiber cell initiation and differentiation. The subsequent transcriptomic analysis identified some phytohormone and secondary metabolism-related genes, which may play important roles in fiber development through histone deacetylation-mediated pathways and other complicated interactions.

## Results

### In vitro TSA treatment of ovule inhibits fiber initiation and earlier elongation

**Fig. 1:**
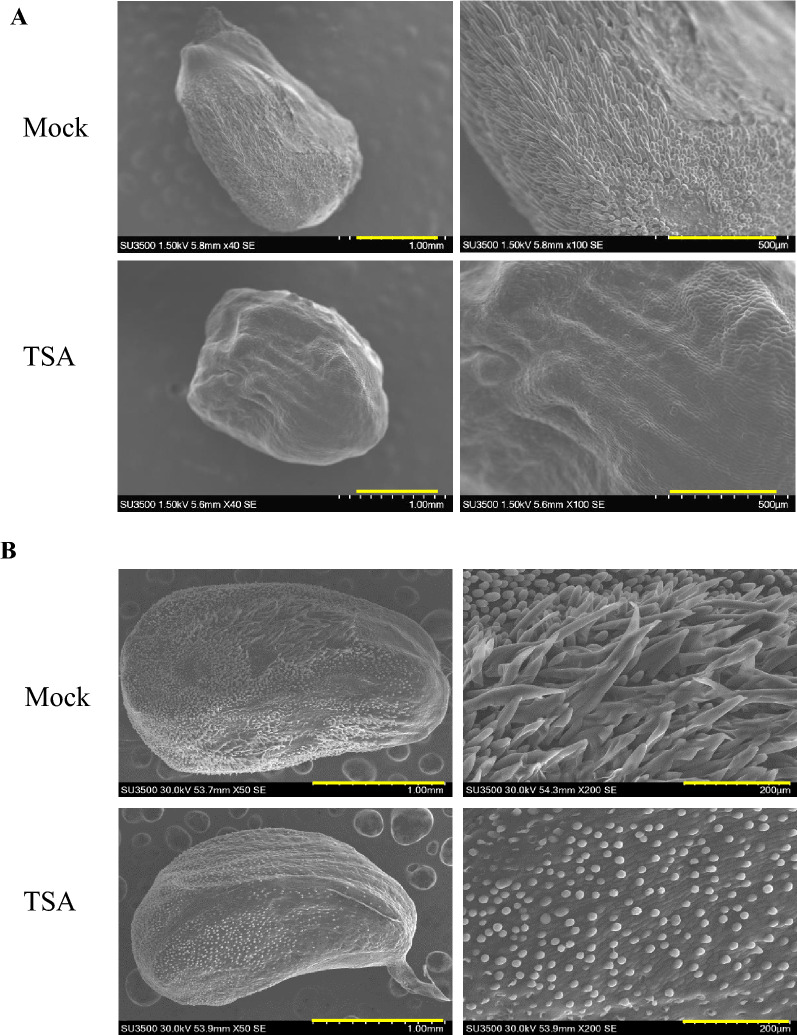
Application of TSA repress fiber cell initiation and elongation in vitro. **A** Ovules of −2 DPA (days post anthesis) were treated with 10 μM TSA for 6 days in vitro, and then the ovules surface were observed and captured by Scanning Electron Microscope. **B** 0 DPA ovules were treated with 10 μM TSA for 2 days in vitro, and the ovules surface were observed and captured by SEM. Bar = 1 mm (intact ovules) and Bar = 200 μm (magnification). The pictures on the right are the magnification of the regions of the pictures on the left

The previous study has shown the important roles of TSA in fiber elongation [6], but the effect of TSA in fiber cell initiation and the underlying mechanisms are still unclear. We used the ovules before and after anthesis (−2 DPA and 0 DPA, respectively) to test the roles of TSA for fiber cell initiation and earlier elongation. The results showed that TSA significantly inhibits the fiber cell initiation as well as the earlier fiber elongation compared with mock in vitro culture (Fig. 1), indicating the important roles of histone deacetylation in fiber cell initiation. The long-term in vitro culture of 0 DPA ovules further confirmed the inhibition of TSA on the fiber elongation (Additional file 5: Fig. S1). Subsequently, the ovules of −2 DPA treated with TSA (10 μM) for 6 days in vitro culture were used for RNA extraction and RNA-Seq with Illumina sequencing platform.

### RNA-Seq of ovules after TSA treatment and data analysis

**Fig. 2:**
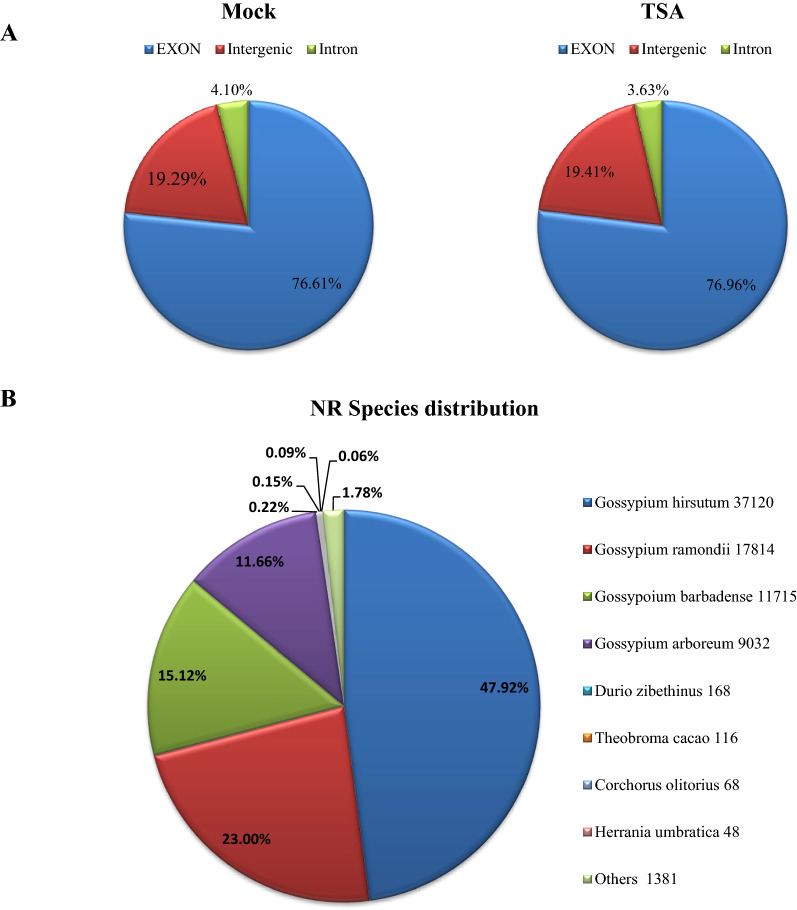
Reads distribution in the genome and natural species distribution of identified genes. **A** All the reads obtained were mapped to the cotton genome. Reads distribution in exon, intergenic, and intron regions were represented for ovules treatment with mock and TSA. **B** A total of 77,555 unigenes were annotated to the Nr protein database. For the species distribution, about 31,720 (47.92%), 17,814 (23%), 11,715 (15.12%), and 9030 (11.66%) unigenes were matched with *Gossypium hirsutum*, *G. ramondii, G. barbadense* and *G. arboretum*. The matched genes are less than 2% in other close species (i.e. *Durio zibethinus*, *Theobroma cacao*, *Corchorus olitorius*, *Herrania umbratica*)

In total, more than 9.01G of clean data was obtained for each sample. The clean sequence reads were used to assemble the transcriptome for each sample by mapping the reads to the cotton reference genome (http://mascotton.njau.edu.cn/info/1054/1118.htm). More than 90.0% of the reads could be mapped to the cotton reference genome, and more than 75%, approximately 20%, and 4% of the mapped reads were mapped to exon, intergenic, and intron regions, respectively (Fig. 2A). The mapped reads from all samples were then remapped to the reference genome and 77,691 unigenes were defined by assembling clean reads with Trinity. All unigenes obtained by transcriptome sequencing were annotated into the NR, KEGG, GO, COG, eggNOG, Swiss-Prot, Pfam, and KOG databases. As a result, 77,555 (NR: 99.82%), 28,174 (KEGG: 36.26%), 57,846 (GO: 74.46%), 26,087 (COG: 33.58%), 70,960 (eggNOG: 91.34%), 55,767 (Swiss-Prot: 71.77%), 59,214 (Pfam: 76.22%) and 41,302 (KOG: 53.16%) unigenes were functionally annotated, respectively. In the NR database, the results showed that *G. hirsutum* had the highest matching degree with the unigene sequence (47.92%), followed by *G. ramondii* (23%), *G. barbadense* (15.12%), and *G. arboretum* (11.66%). The matched genes are less than 2% in other close species (i.e. *Durio zibethinus*, *Theobroma cacao*, *Corchorus olitorius*, *Herrania umbratica*), and 1.78% of the unigenes did not match the protein sequences of other species (Fig. 2B), indicating the accuracy of RNA-Seq and some specificity of the cotton genes.

**Fig. 3:**
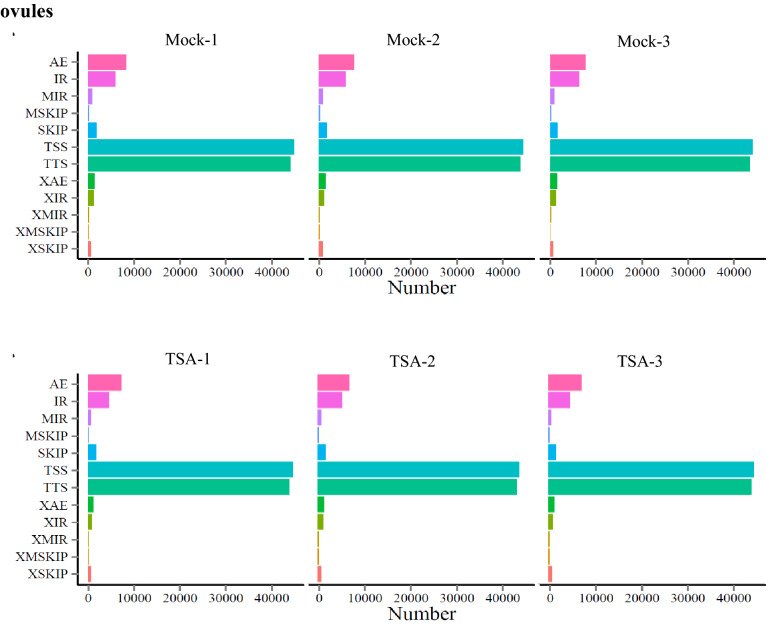
Alternative splicing analysis of transcripts identified in Mock and TSA treated ovules. In the six libraries including mock and TSA treated, alternative splicing of the transcripts was analyzed. In total, 12 kinds of AS events were defined as follows, and the results showed that TSS and TSS are the dominant AS events identified. (1) TSS: Alternative 5’first exon (transcription start site); (2) TTS: Alternative 3’ last exon (transcription terminal site); (3) SKIP: Skipped exon (SKIP_ON, SKIP_OFF pair); (4) XSKIP: Approximate SKIP (XSKIP_ON, XSKIP_OFF pair); (5) MSKIP: Multi-exon SKIP (MSKIP_ON, MSKIP_OFF pair); (6) XMSKIP: Approximate MSKIP (XMSKIP_ON, XMSKIP_OFF pair); (7) IR: Intron retention (IR_ON, IR_OFF pair); (8) XIR: Approximate IR (XIR_ON, XIR_OFF pair); (9) MIR: Multi-IR (MIR_ON, MIR_OFF pair); (10) XMIR: Approximate MIR (XMIR_ON, XMIR_OFF pair); (11) AE: Alternative exon ends (5′, 3′, or both); (12) XAE: Approximate AE. X: exact boundary match for SKIP, approximate (simple exon overlap) for XSKIP; An exon skipping event as a pair between an exon containing (‘on’) splice form and an exon-skipping (‘off’) splice

Alternative splicing (AS), generating distinct mRNA species and non-coding RNAs (ncRNAs) from one primary transcript, functions as an additional regulatory mechanism for gene expression and function after transcription. This endows AS with the importance of bringing protein and function diversity from a definite gene [31]. The conserved AS events were identified and there were 12 types of AS events found across all the samples. Two types of ASs at the 5' first exon (TSS, transcription start site) and 3' last exon (TTS, transcription terminal site) are the most, followed by alternative exon ends (AE) at the 5' end, 3' end, or both and intron retention (IR) (Fig. 3), which indicates the various and complicated regulatory mechanisms of gene post-transcription.

### Analysis of differentially expressed genes in ovules after TSA treatment

**Fig. 4:**
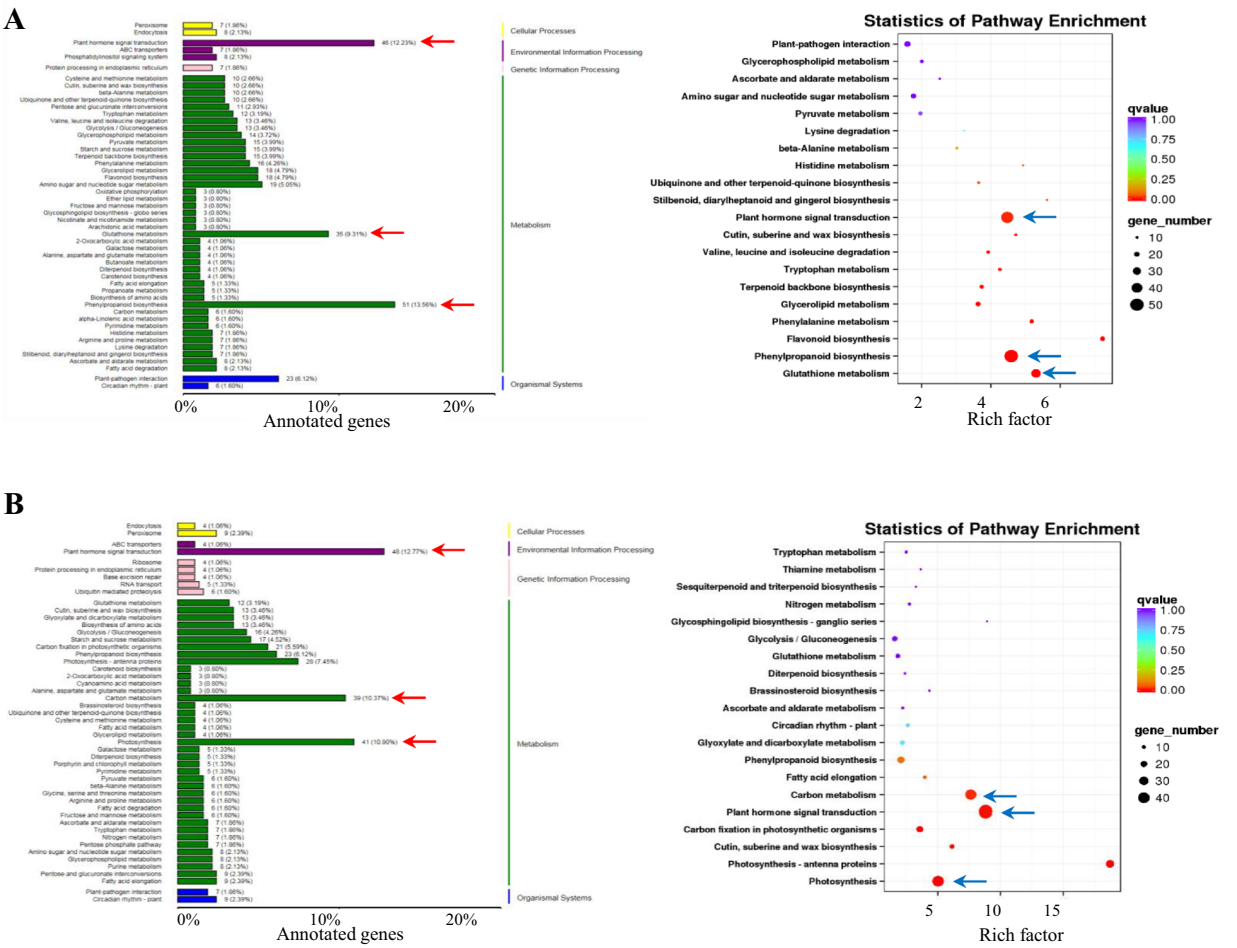
KEGG analysis of up-and down-regulated genes after TSA treatment. KEGG analysis was performed and the differentially expressed genes were classified into five categories: cell process, environmental information processing, genetic information processing, metabolism, and organismal systems. The top 50 pathways were presented here. **A** Among the up-regulated genes, metabolism accounted for the largest proportion of all the categories. Furthermore, the plant hormone signal transduction (46 genes), glutathione metabolism (35 genes), and phenylpropanoid biosynthesis pathways (51 genes) showed the most genes (red arrows). The statistical analysis of enrichment pathways showing the lowest q-value of plant hormone signal transduction, carbon metabolism, and phenylpropanoid biosynthesis pathways also indicated the reliable enrichment significance (blue arrows). **B** In the down-regulated genes, plant hormone signal transduction (48 genes), photosynthesis (41 genes), and carbon metabolism pathways (39 genes) showed the most genes (red arrows). The statistical analysis of the three enrichment pathways showed their lowest q-value and the reliable significance of enrichment pathways (blue arrows). Statistics of pathway enrichment of DEGs were analyzed using Rich Factor and *q*-value. Circle size indicates the gene numbers of the different pathways, which is the ratio of differentially expressed gene numbers to all gene numbers in this pathway term. A larger size means a greater number of genes. *Q*-value is the corrected *p*-value ranging from 0 to 1, and a lower *q*-value means greater intensiveness

To explore the underlying mechanisms of TSA inhibiting fiber initiation, DEG were identified between mock and TSA-treated ovules. The MA plots showed that most genes did not change in transcription level (black plots); the numbers of up-regulated and down-regulated genes were similar with significant alteration of transcription (red and green plots) (Additional file 5: Fig. S2). In total, 4209 genes were identified with 2025 genes up-regulated and 2184 down-regulated in response to TSA. Among which, 4196 genes were annotated including 3980 known genes and 216 new genes (annotated), as well as 13 unknown genes (without annotation and locus) (Table 1 and Additional file 1: Table S1). To illuminate the potent causal pathways and key genes, KEGG enrichment pathway analysis was performed. The up-regulated genes were classified into 95 pathways among five categories: cell process, environmental information processing, genetic information processing, metabolism, and organismal systems. Of these five categories, the top 50 pathways were presented (Fig. 4A). Metabolism accounted for the largest proportion amongst the five categories. Furthermore, the plant hormone signal transduction (46 genes) and phenylpropanoid biosynthesis pathways (51 genes) showed the most genes observed. The statistical analysis of enrichment pathways showed the lowest q-value (red arrow) and their reliable enrichment significance (Fig. 4A). The down-regulated genes were classified into 97 pathways among five categories, and the top 50 pathways were represented. The plant hormone signal transduction (48 genes), photosynthesis (41 genes), and carbon metabolism pathways (39 genes) showed the most genes observed. The statistical analysis of the three enrichment pathways showed their lowest q-value (red arrow) and their reliable enrichment significance (Fig. 4B). The above results suggest that many genes related to plant hormone signal transduction may play important roles in the initial ovule and fiber development mediated by histone deacetylation. Moreover, up-regulated DEGs were also largely enriched in phenylpropanoid biosynthesis (Fig. 4A); while carbon metabolism and photosynthesis-related genes were enriched in the down-regulated genes (Fig. 4B), providing evidence that histone deacetylation functions upstream of diverse metabolism pathways in order to regulate fiber initiation.

**Table 1:** The summary of differentially expressed genes in the ovules after TSA treatment

Mock_vs_TSA	Gene numbers		
Total identified gene number	77,691		
Total DEG Number	4209	2025 (up-regulated)	2184 (down-regulated)
Annotated genes	4196	216 new genes (without loci)	3980 known genes (with defined loci)
Unknown new gene	13

### Phytohormones signal transduction play important roles in fiber initiation downstream of histone deacetylation

**Fig. 5:**
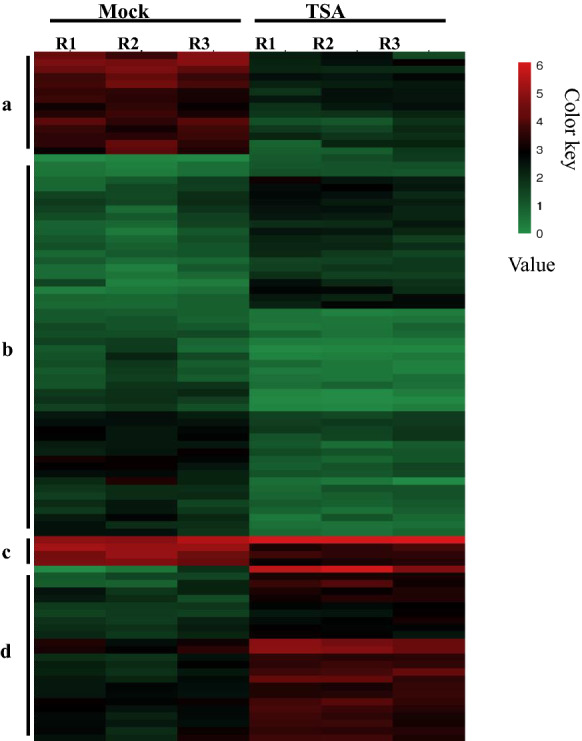
Heatmap of the 94 DEGs associated with hormonal pathways after TSA treatment. The heatmap of FPKM (fragments per kilobase of transcript per million reads mapped) of 94 DEGs associated with hormone signal transduction in three biological replicates of Mock and TSA treatment ovules. The DEGs were then divided into four groups (**a**–**d**) according to the expression profiles in ovules of mock and TSA treatments. **a** The genes significantly up-regulated in response to TSA treatment; **b** the genes that showed a weak difference with less FPKM values; **c** the genes that showed a weak difference with more FPKM values; **d** the genes significantly down-regulated in response to TSA treatment. Log10-transformed (FPKM + 1) expression values were used to create the heat map. The red and green colors represent the higher and lower relative abundance of each transcript in each sample, respectively

In total, 94 up-and down-regulated DEGs associated with hormone signal transduction were used to draw the heat map with the FPKM values in all the samples (Fig. 5 and Additional file 2: Table S2). The resulting heat map divided the DEGs into four classifications a, b, c, and d. In class a, the genes were significantly up-regulated in response to TSA treatment, while genes were significantly down-regulated in response to TSA treatment in class d. In classes b and c, the difference in transcription level is significantly less weak in ovules of Mock compared with TSA treatments. The FPKM values of each gene in class b were less than 6, while those in class c were greater than 8 (Additional file 3: Table S3).

To understand the detailed hormonal pathways, we analyzed the up-and down-regulated genes. It is shown that most of the down-regulated genes are related with the auxin pathway. This includes small auxin up-regulated RNA (SAUR), INDOLE-3-ACETIC ACID INDUCIBLE (IAA), AUXIN RESPONSE FACTOR (ARF), auxin receptor, and genes associated with IAA synthesis and homeostasis which accounts for about 65.4% of the down-regulated genes, indicating the positive role of auxin in fiber initiation (Table 2). Interestingly, among the up-regulated members, auxin-related genes (e.g., small auxin up-regulated RNA and auxin influx carrier), The JASMONATE-ZIM DOMAIN (JAZ)-like factors associated with JASMONIC ACID (JA), ABA associated genes (e.g., ABA receptors, signaling factors) account for 32.6%, 30.4%, and 15.2%, respectively. Furthermore, three GA receptors also showed clear up-regulated expression (Table 3). Auxin plays important positive roles in fiber initiation downstream of histone deacetylation, whereas some small auxin up-regulated RNA encoding genes and auxin influx carriers also play negative roles in fiber initiation. ABA pathways and JA inhibitory factor JAZ2 play negative roles in fiber initiation downstream of histone deacetylation similar to previous studies [32–34], which suggest that histone deacetylation plays a vital role in fiber initiation and earlier elongation through regulating various phytohormone signaling pathways.

**Table 2:** Summary and identification of the down-regulated genes associated with phytohormone pathways in response to TSA treatment

Gene ID	Gene name	Annotation	Regulated type	Fold change (Log2)	Associated pathway
Gh_A10G1020	GhIAA3_A10	INDOLE-3-ACETIC ACID INDUCIBLE 3	Down	− 2.0372	Auxin
Gh_D02G0405	GhIAA4_D02	INDOLE-3-ACETIC ACID INDUCIBLE 4	Down	− 2.2086	Auxin
Gh_A09G1945	GhIAA4_A09		Down	− 2.7903	Auxin
Gh_A02G0340	GhIAA4_A02		Down	− 1.5551	Auxin
Gossypium_hirsutum _newGene_23351	GhIAA4_N		Down	− 1.0348	Auxin
Gh_D01G0947	GhIAA4_D01		Down	− 2.7321	Auxin
Gh_A02G0341	GhIAA14_A02	INDOLE-3-ACETIC ACID INDUCIBLE 14	Down	− 1.1472	Auxin
Gh_A11G1164	GhIAA16_A11	INDOLE-3-ACETIC ACID INDUCIBLE 16	Down	− 2.6413	Auxin
Gh_D05G0472	GhIAA18_D05	INDOLE-3-ACETIC ACID INDUCIBLE 18	Down	− 3.8157	Auxin
Gh_A05G0356	GhIAA18_A05		Down	− 1.5554	Auxin
Gh_A06G1644	GhIAA19_A06	INDOLE-3-ACETIC ACID INDUCIBLE 19	Down	− 3.9605	Auxin
Gh_D10G1512	GhIAA19_D10		Down	− 6.1937	Auxin
Gh_A05G1607	GhIAA24_A05	INDOLE-3-ACETIC ACID INDUCIBLE 24	Down	− 1.319	Auxin
Gh_D05G1792	GhIAA24_D05		Down	− 1.1334	Auxin
Gh_D11G1228	GhIAA29_D11	INDOLE-3-ACETIC ACID INDUCIBLE 29	Down	− 1.8453	Auxin
Gh_D11G0514	GhGH3.1_D11	ENCODING A PROTEIN SIMILAR TO IAA-AMIDO SYNTHASES GH3.1	Down	− 2.4801	Auxin
Gh_A11G1077	GhGH3.3_A11	ENCODING AN PROTEIN SIMILAR TO IAA-AMIDO SYNTHASES GH3.3	Down	− 1.6851	Auxin
Gh_D13G0668	GhGH3-10_D13	ENCODING AN PROTEIN SIMILAR TO IAA-AMIDO SYNTHASES GH3-10	Down	− 2.692	Auxin
Gh_A11G0443	GhGH3-10_A11		Down	− 2.0607	Auxin
Gh_A13G0480	GhGH3-10_A13		Down	− 1.2652	Auxin
Gh_D02G2216	GhSAUR1_D02	SMALL AUXIN UP-REGULATED RNA11	Down	− 2.3625	Auxin
Gh_D12G0291	GhSAUR12_D12	SMALL AUXIN UPREGULATED RNA12	Down	− 2.747	Auxin
Gh_A13G0578	GhSAUR48_A13	SMALL AUXIN UPREGULATED RNA48	Down	− 1.0704	Auxin
Gh_D12G0289	GhSAUR52_D12	SAUR52, SMALL AUXIN UPREGULATED RNA52	Down	− 1.0695	Auxin
Gh_D10G0616	GhPAP1_D10	PURPLE ACID PHOSPHATASE 1	Down	− 1.998	Auxin
Gh_A02G1156	GhPAP2_A02	PURPLE ACID PHOSPHATASE 2	Down	− 1.7051	Auxin
Gh_A05G1076	GhPAP2_A05		Down	− 1.289	
Gh_A03G1804	GhLAX2_A03	LIKE AUXIN RESISTANT 2	Down	− 2.3804	Auxin
Gh_D01G1617	GhLAX2_D01		Down	− 3.069	Auxin
Gh_A01G1374	GhLAX2_A01		Down	− 2.3246	Auxin
Gh_D09G1378	GhTIR1_D09	AUXIN RECEPTOR, TRANSPORT INHIBITOR RESPONSE 1	Down	− 3.9574	Auxin
Gh_D09G2152	GhTIR1_D09		Down	− 2.3805	Auxin
Gh_A12G1016	GhARF11_A12	AUXIN RESPONSE FACTOR 11	Down	− 1.6769	Auxin
Gh_A07G1254	GhARF18_A07	AUXIN RESPONSE FACTOR 18	Down	− 1.345	Auxin
Gh_Sca005214G02	GhARR2_S	RESPONSE REGULATOR 2	Down	− 1.8818	Cytokinin
Gh_D05G2656	GhARR7_D05	RESPONSE REGULATOR 7	Down	− 1.2673	Cytokinin
Gh_A11G2364	GhAHP5_A11	HISTIDINE-CONTAINING PHOSPHOTRANSFER FACTOR 5	Down	− 1.0153	Cytokinin
Gh_A11G1389	GhAHK2_A11	HISTIDINE KINASE 2	Down	− 1.151	Cytokinin
Gh_D01G1042	GhCYCD3;2_D01	CYCLIN D3;2	Down	− 2.2107	Cytokinin
Gh_D05G0138	GhTGA7_D05	TGACG SEQUENCE-SPECIFIC BINDING PROTEIN 7	Down	− 1.4757	SA
Gh_A02G0775	GhTGA7_A02		Down	− 2.0558	SA
Gh_D12G1679	GhTGA8_D12	TGACG SEQUENCE-SPECIFIC BINDING PROTEIN 8	Down	− 3.7153	SA
Gh_A09G0903	GhTGA9_A09	TGACG SEQUENCE-SPECIFIC BINDING PROTEIN 9	Down	1.3068	SA
Gh_A07G1202	GhPIF3_A07	PHYTOCHROME INTERACTING FACTOR 3	Down	− 1.1952	GA
Gh_D13G1847	GhGID1C_D13	GA INSENSITIVE DWARF1C	Down	− 1.7948	GA
Gh_A05G0308	GhHAB1_A05	HYPERSENSITIVE TO ABA1, NEGATIVE IN ABA SIGNALING	Down	− 1.0137	ABA
Gh_D01G1881	GhBAK1_D01	BRI1-ASSOCIATED RECEPTOR KINASE	Down	− 1.1379	BR
Gh_D05G2975	GhJAI3_D05	JASMONATE-INSENSITIVE 3	Down	− 1.8414	JA

**Table 3:** Summary and identification of the up-regulated genes associated with phytohormone pathways in response to TSA treatment

Gene ID	Gene name	ANNOTATION	Regulated type	Fold change (Log2)	Associated pathway
Gh_A01G1955	GhAUX1_A01	AUXIN RESISTANT 1	Up	1.26685	Auxin
Gh_A05G3362	GhGH3.17_A05	ENCODING A PROTEIN SIMILAR TO IAA-AMIDO SYNTHASES GH3.17	Up	1.51936	Auxin
Gossypium_hirsutum_newGene_7580	GhGH3.6_N	ENCODING A PROTEIN SIMILAR TO IAA-AMIDO SYNTHASES GH3.17	Up	1.49656	Auxin
Gh_D08G1987	GhGH3.7_D08	ENCODING A PROTEIN SIMILAR TO IAA-AMIDO SYNTHASES GH3.17	Up	1.48741	Auxin
Gh_A06G0351	GhLAX3_A06	LIKE AUX1 3	Up	1.23712	Auxin
Gh_A03G1990	GhSAUR34_A03	SMALL AUXIN UP-REGULATED RNA34	Up	1.10734	Auxin
Gh_D03G1541	GhSAUR34_D03		Up	1.33354	Auxin
Gh_D08G1114	GhSAUR31_D08	SMALL AUXIN UP-REGULATED RNA31	Up	1.02448	Auxin
Gh_A12G2237	GhSAUR31_A12		Up	2.45374	Auxin
Gh_D13G2504	GhSAUR49_D13	SMALL AUXIN UP-REGULATED RNA49	Up	1.49511	Auxin
Gh_A03G1766	GhSAUR50_A03	SMALL AUXIN UP-REGULATED RNA50	Up	2.5748	Auxin
Gh_D12G2759	GhSAUR55_D12	SMALL AUXIN UP-REGULATED RNA55	Up	1.99109	Auxin
Gh_D08G2457	GhSAUR72_D08	SMALL AUXIN UP-REGULATED RNA55	Up	1.0073	Auxin
Gh_D08G1506	GhSAUR59_D08	SMALL AUXIN UP-REGULATED RNA55	Up	1.68807	Auxin
Gh_D02G2199	GhSAUR8_D02	SMALL AUXIN UP-REGULATED RNA55	Up	2.19574	Auxin
Gh_A08G2199	GhJAZ1_A08	JASMONATE-ZIM-DOMAIN PROTEIN 1	Up	1.82836	JA
Gh_D08G2564	GhJAZ1_D08		Up	1.7424	JA
Gh_A05G0260	GhJAZ1_A05		Up	1.79396	JA
Gh_D05G0352	GhJAZ1_D05		Up	1.93514	JA
Gh_D06G0810	GhJAZ1_D06		Up	1.09472	JA
Gh_D02G1776	GhJAZ10_D02	JASMONATE-ZIM-DOMAIN PROTEIN 10	Up	2.0817	JA
Gh_D01G0196	GhJAZ10_D01		Up	1.39144	JA
Gh_A03G1341	GhJAZ10_A03		Up	2.58634	JA
Gh_A01G0153	GhJAZ10_A01		Up	5.02041	JA
Gh_A05G1155	GhJAZ6_A05	JASMONATE-ZIM-DOMAIN PROTEIN 6	Up	1.10218	JA
Gh_D10G0531	GhJAZ6_D10		Up	1.29964	JA
Gh_A12G2172	GhMYC4_A12	A BHLH TRANSCRIPTIONAL REGULATOR INTERACTING WITH JAZ	Up	2.22509	JA
Gh_D02G0731	GhCOI1_D02	CORONATINE INSENSITIVE 1	Up	1.71994	JA
Gh_D01G1903	GhTGA10_D01	TGACG SEQUENCE-SPECIFIC BINDING PROTEIN 10	Up	1.86541	SA
Gh_A12G0274	GhPR1_A12	PATHOGENESIS-RELATED GENE 1	Up	5.6104	SA
Gh_A10G0769	GhBZIP_A10	BASIC LEUCINE-ZIPPER	Up	1.53952	SA
Gh_A12G2380	GhAHG1_A12	ABA-HYPERSENSITIVE GERMINATION 1	Up	1.23584	ABA
Gh_D10G2388	GhPYL5_D10	PYRABACTIN RESISTANCE 1-LIKE 5	Up	1.73882	ABA
Gh_A10G2142	GhPYR5_A10		Up	1.10077	ABA
Gh_D11G0290	GhPYR1_D11	PYRABACTIN RESISTANCE 1-LIKE 1	Up	1.26703	ABA
Gh_A06G1418	GhPYR6_A06	PYRABACTIN RESISTANCE 1-LIKE 6	Up	2.40182	ABA
Gh_A05G1922	GhSRK2C_A05	SNF1-RELATED PROTEIN KINASE 2C	Up	1.12806	ABA
Gh_D11G3472	GhSRK2E_D11	SNF1-RELATED PROTEIN KINASE 2E	Up	1.53833	ABA
Gh_A08G1649	GhGID1_A08	GA INSENSITIVE DWARF1	Up	1.44491	GA
Gh_D11G1242	GhGID1B_D11	GA INSENSITIVE DWARF1B	Up	1.19774	GA
Gh_D08G1981	GhGID1B_D08		Up	1.55219	GA
Gh_D13G0548	GhBAK1_D13	BRI1-ASSOCIATED RECEPTOR KINASE	Up	2.87103	BR
Gh_A01G0972	GhBKI_A01	BRI1 KINASE INHIBITOR 1	Up	1.94267	BR
Gh_A10G0769	GhBZIP_A10	BASIC LEUCINE-ZIPPER	Up	1.53952	SA
Gh_D02G0430	GhERF1_D02	ETHYLENE RESPONSE FACTOR 1	Up	2.85319	Ethylene
Gh_Sca115107G01	GhERF1B	ETHYLENE RESPONSE FACTOR 1B	Up	3.76382	Ethylene
Gh_A01G0218	GhARR2_A01	RESPONSE REGULATOR 2	Up	1.2164	cytokinin

**Fig. 6:**
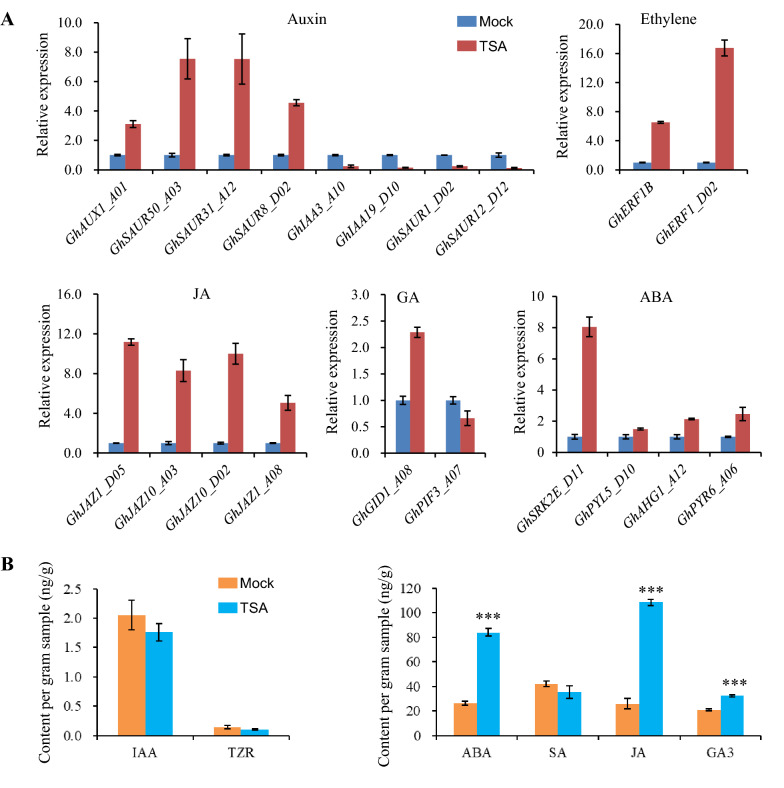
Verification of some key genes transcription associated with TSA and determination of phytohormones content in response to TSA during fiber initiation. **A** The expression profiles of key genes associated with phytohormone pathways were determined in the mock and TSA-treated ovules by RT-qPCR. All the expression levels were normalized to UBQ7, the gene was then expressed as a ratio relative to mock, which was set to a value of 1. Primers are listed in the Additional file 4: Table S4. **B** Different plant hormone levels were determined in TSA-treated ovules of − 1 DPA culture for 5 days. Error bars indicated SD (standard deviation) of three biological replicates

Moreover, some key genes' transcription expression in phytohormone pathways were validated in TSA-treated ovules with quantitative PCR (Fig. 6A). The results showed that some genes such as *GhIAA3_A1*0, *GhIAA19_D10*, *GhSAUR1_D02,* and *GhSAUR12_D12* were significantly down-regulated, while some genes, including *GhAUX1_A01*, *GhSAUR50_A03*, *GhSAUR31_A12*, and *GhSAUR8_D02* were clearly up-regulated in the auxin signaling pathway. Two ethylene response factors *GhERF1B* and *GhERF1_D02*, as well as four negative regulators in the JA pathway- JAZ protein-coding genes *GhJAZ1_A08/D05* and *GhJAZ10_A03/D02* showed obvious up-regulation. Two factors in the gibberellin (GA) pathways *GhGID1_A08* and *GhPIF3_A07* showed up-and down-regulation respectively in TSA-treated ovules. Four abscisic acids (ABA) signaling genes *GhSRK2E_D11*, *GhPYL5_D10*, *GhPYL6_A06,* and *GhAHG1_A12* showed significant up-regulation in response to TSA treatment. Simultaneously, we further detected different hormone contents in TSA-treated ovule. Compared to mock, TSA significantly promoted the accumulation of ABA, JA and GA_3_, while inhibiting the synthesis of IAA and not influencing SA and TZA contents significantly (Fig. 6B). All the results point to the regulation of different plant hormones by histone deacetylation in fiber initiation and elongation.

**Fig. 7:**
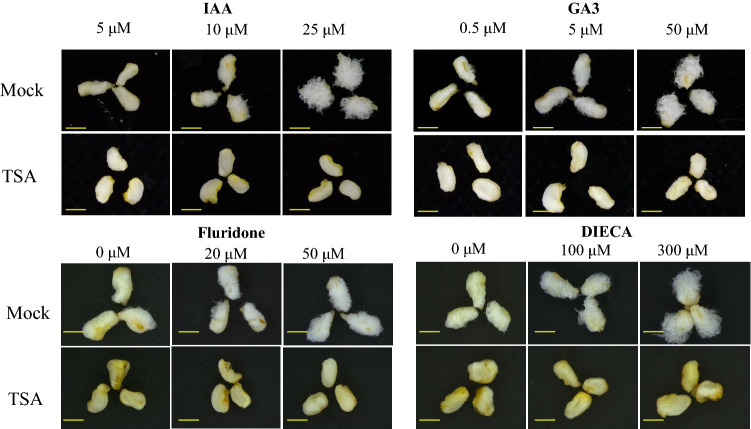
TSA inhibits the promotion on fiber initiation of IAA and GA_3_ and the inhibitors of ABA and MeJA. Ovules of −1 DPA (days post anthesis) were cultured in BT medium supplemented with 10 μM DMSO (mock) or TSA for 5 days in vitro, and these media contained different concentration gradients of IAA (5,10,25 μM), GA_3_ (0.5, 5, 50 μM), Fluridone (0, 20, 50 μM) and DIECA (0, 100, 300 μM), Bar = 1 mm

To verify the important role of phytohormones in regulating fiber initiation and elongation, the ovules of −2 DPA were incubated in modified BT- mediums without GA_3_ or IAA, supplemented with 20 μM ABA or Methyl Jasmonate (MeJA) of different concentrations (0.05, 0.5, and 5 μM) for 7 or 14 days in vitro. The results show that both fiber initiation and development are severely inhibited when GA3 or IAA is deficient, especially for GA3. Similarly, no fiber initiated after 7 days of culture, and little fiber was present after 14 days in response to 20 μM ABA (Additional file 5: Fig. S3A). However, in the medium containing MeJA, the fibers were longer than that in the mock medium. Among the different concentrations, 0.05 μM MeJA promoted fiber elongation most effectively (Additional file 5: Fig. S3B). These results demonstrate the positive regulation of GA, auxin and MeJA, and the inhibitory effect of ABA on fiber initiation and development. To further verify the effect of TSA on these hormones during fiber development, we supplemented different concentration gradients of IAA (5, 10, 25 μM), GA_3_ (0.5, 5, 50 μM), Fluridone (an inhibitor of ABA biosynthesis, 0, 20, 50 μM), and sodium diethyldithiocarbamate (DIECA, an inhibitor of jasmonic acid synthesis, 0, 100, 300 μM) into the BT medium containing 10 μM TSA. After 5 days, we observed that IAA, GA_3_, Fluridone, and DIECA promoted fiber elongation dependent on the concentrations in BT medium without TSA, while not restoring any inhibitory phenotype of TSA on fiber development when 10 μM TSA was supplied (Fig. 7). These results suggest that TSA inhibits fiber initiation by regulating different phytohormone pathways such as IAA, GA_3_, ABA and MeJA.

## Discussion

Cotton fiber is one of the most important products worldwide and the model for cell differentiation and elongation study [35]. Lots of research has been carried out to clarify the underlying mechanism of fiber development, and many important genes have been found to be involved in the different pathways to regulate fiber cell initiation, elongation, secondary cell wall deposition, and maturation [6, 32, 36]. Among the pathways involved in fiber development, histone (de)acetylation is one of the important epigenetic modifications found to be playing a crucial role. One such role is observed by the *HDA5* gene, which was identified as a key histone deacetylase in fiber initiation [6, 37]. In total, eighteen HATs and thirty HDACs have been identified from *G. hirsutum* [6, 38]. However, the detailed roles of different HATs or HDACs and the associated regulatory networks remain unclear.

### HDACi-TSA represses the fiber initiation and earlier elongation significantly

Due to the important roles of histone acetylation in eukaryotes development, HDACi, which can repress histone deacetylation, has been applied in eukaryote development studies and disease therapies [5, 21, 27–29]. The application of TSA in the In vitro culture of ovules has also showed its repressive role in fiber differentiation [6]. Here, we used TSA in the in vitro culture of ovules before and after anthesis, and the results showed that TSA inhibits the fiber cell initiation, as well as the primary elongation (Fig. 1). These findings indicate that histone acetylation plays a significant role in fiber development and moved us to explore the underlying mechanisms, thereby an RNA-Seq was made using the ovules treated with TSA and Mock.

### DEGs analysis in the ovule after TSA treatment through RNA-Seq

From the libraries, a total of 77,691 genes were identified, which is very close to the total genes in the entire genome and indicates a greater library quality. Analysis of the mapped sequence reads showed that about 23% of reads were of the mapped intergenic and intron regions, which implied that some novel genes were identified from the libraries of ovules. The alternative splicing analysis of transcripts displayed that TSS and TTS types are the most AS types in cotton, which is what has been observed in previous studies [39–41]. In *Arabidopsis* and rice, ‘intron retention’ was the prevalent type [40, 42, 43]. By contrast, ‘exon skipping’ is the most dominant pattern in humans and yeast [39, 41], which proposed that AS varies during eukaryote evolution.

The DEGs in response to TSA treatment were identified to illuminate the underlying mechanism of fiber initiation involved by histone deacetylation. In total, 4209 DEGs including 229 new genes consisting of 216 annotated genes and 13 genes without annotation and locus information were found, which implies that the public genome data is still not perfect and more advanced technology of assembly is necessary. A KEGG analysis was then operated and the results showed that plant hormone signal transduction, phenylpropanoid biosynthesis, and glutathione metabolism are the most enriched groups in up-regulated genes. In contrast, plant hormone signal transduction, carbon metabolism, and photosynthesis are the most enriched groups in down-regulated genes (Fig. 4). Phenylpropanoid pathway is associated with flavonoid biosynthesis, which competes with the fatty acid pathway for malonyl-CoA [44, 45], and the fatty acid metabolism mediates fiber development [46, 47]. Moreover, a previous study demonstrated that flavonoid naringenin is negatively associated with fiber development and that the flavonoid metabolism mediated by flavanone 3-hydroxylase is important in fiber development [48], which is in agreement with our findings and provides clues for the underlying mechanism associated with flavonoid biosynthesis regulated by histone deacetylation. Glutathione (GSH), an α-amino acid and a tripeptide, functions as a molecule that protects cells against oxidation through providing the cell with its reducing milieu which maintains various cellular components including enzymes in a reduced state. Furthermore, glutathione also functions as a storage and transport form of cysteine moieties [49, 50]. Cysteine is an important intermediate of sulfur metabolism in plants and functions as the reduction state of sulfite, which is reduced by the enzyme APS reductase and the cysteine synthase complex, as a result of the interaction of the enzymes serine acetyltransferase and *O*‐acetylserine‐(thiol)‐lyase, in order to control flux through the pathway [51–53]. Therefore, the up-regulated phenylpropanoid biosynthesis and glutathione metabolism indicate that the fatty acid pathway and sulfur metabolism are involved in the fiber initiation regulation which is at least partially dependent on histone acetylation, which supports the previous studies as well as provides novel clues for fiber cell development studies [46, 47].

Carbon metabolism and photosynthesis are closely related and control the energy production, transport, and application in the life cycle of plants [54–56]. The repressed carbon metabolism and photosynthesis after TSA treatment led to attenuated energy metabolism in the ovules, which is consistent with the retarded fiber development. More interestingly, the phytohormone signal transduction was enriched in both up-and down-regulated pathways, indicating that different hormones may be involved in the fiber development downstream of histone deacetylation, which is also in line with the previous studies [36, 57, 58].

### Histone deacetylation functions upstream of the phytohormones to regulate fiber development

Many studies have shown that phytohormones such as auxin, GA, ethylene, cytokinin (CK), brassinosteroids (BR), and ABA play important roles in fiber development [35, 36, 57, 59]. Our RNA-Seq results supported the roles of phytohormones and provided some potential regulatory mechanisms associated with histone acetylation modification. The intensive analysis of the hormone pathway revealed that 49 auxin pathway-related genes showed differential expression. This included 34 up-regulated and 15 down-regulated genes after TSA treatment, illustrating the dominant role of auxin in the fiber development of cotton which supports the pivotal role of auxin in fiber cell initiation [60]. In *Arabidopsis*, HDA6, HDA9, and HDA19 have shown the regulatory roles in transcription expression of *AUX1* and some *ARF*s through histone deacetylation modification which regulate the auxin pathway, seed germination, and valve cell elongation [61, 62]. Some *IAA*s are also regulated by histone deacetylation to respond to aberrant ambient light and temperature of the plant [63, 64]. Here, *GhAUX1_A01* and some *GhSAUR*s (e.g., *GhSAUR50_A03*, *GhSAUR31_A12*, and *GhSAUR8_D02*) exhibited up-regulation indicating the potential auxin import and accumulation of non-fiber cells. Inversely, the down-regulation of *GhIAA*s and *GhSAUR*s and resulting decreased IAA content, hints at the conceivable repression of auxin signal in fiber cells, which inhibits the fiber initiation downstream of histone deacetylation. Interestingly, auxin has been shown to be the key role in the fiber cell initiation through the proper spatiotemporal accumulation and distribution, in which the auxin efflux carrier PIN3 plays an important positive role [65]. Moreover, CK inhibits the non-fiber-localized protein GhPIN3a and damages the normal auxin concentration gradient, negatively impacting fiber cell initiation [58, 60]. All this suggests that different auxin pathway genes involved in auxin synthesis, transport, and signaling play distinct roles in fiber development, and auxin partially promotes fiber cell initiation through histone acetylation regulation.

As an important hormone in plant development and abiotic stress tolerance, ABA also exhibits a negative role in fiber cell development [32, 34, 35, 57], although the underlying mechanism is ambiguous. Here, all the identified ABA pathway genes encoding receptors and signaling factors except *HAB1* (a negative regulator in ABA signaling), as well as ABA content were all significantly up-regulated leading to its negative function in fiber initiation and offering the potential epigenetic mechanism upstream of the ABA pathway. JAZ2 is a key negative regulator in cotton fiber initiation [32] and some of its homologues (e.g., *GhJAZ1_A08/D05* and *GhJAZ10_A03/D02*) showed higher expression after TSA treatment, which was consistent with the increased JA accumulation and supported the negative role of JAZ in fiber initiation and providing clues between histone deacetylation and JA pathway in fiber initiation. In contrast, the GA receptor gene *GhGID1_A08* displayed a significant up-regulation, matching the increased GA content in response to TSA. These results implied that TSA might suppress GA signaling through some genes such as *GhPIF3_A07* in fiber initiation, and also raised some clues about the relationship between phytohormones and epigenetic modifications in fiber development. In order to further confirm the effect of histone deacetylation on fiber development, we used another histone deacetylation inhibitor-diallyl disulfide (DADS) [28], which inhibited fiber initiation and even considerably affected ovule development similar to TSA (Additional file 5: Fig. S4). However, the relationship between phytohormones and different secondary metabolism/carbon metabolisms still needs much more work.

## Conclusions

In short, we highlighted that histone deacetylation inhibitor-trichostatin A (TSA) represses the fiber cell initiation and elongation of cotton in in vitro cultures, indicating the crucial roles of histone deacetylation in cell differentiation and development. RNA-Seq revealed that there are 2025 up-regulated genes associated with plant hormone signal transduction and phenylpropanoid biosynthesis, and 2184 down-regulated genes associated with plant hormone signal transduction, photosynthesis, and carbon metabolism, in response to TSA treatment. Further studies showed that TSA repressed auxin, GA, and JA signaling, while promoting ABA signaling in order to inhibit fiber cell initiation and earlier elongation. Phytohormones play versatile roles in plant development, including the development of fiber. Our work demonstrated that histone deacetylation contributes a great deal to the signaling of different phytohormones in fiber initiation, which is very helpful for us to understand the detailed molecular mechanisms of phytohormone pathway regulation and fiber development.

## Materials and methods

### In vitro culture and scanning electron microscopy (SEM) analysis of cotton ovules

Cotton cultivar ZM24 (also known as CCRI24), obtained from the Institute of Cotton Research, the Chinese Academy of Agricultural Sciences were grown under field conditions in Zhengzhou China for the following study [66]. Flowers were harvested at − 2 and 0 days post-anthesis (DPA), and ovaries were surface sterilized by using 75% ethanol. Ovules were carefully dissected from the ovaries under sterile conditions and immediately floated on liquid BT media supplemented with optimized concentrations of phytohormones including IAA (Sigma-Aldrich, I2886), GA3 (Sigma, G7645), ABA (Sigma, A1049), MeJA (Sigma-Aldrich, 392707) as well as Fluridone (A inhibitor for ABA synthesis, Supelco, 45511) and DIECA (A inhibitor for MeJA synthesis, Sigma-Aldrich, 228680). In addition, various concentrations of TSA and DADS (Sigma-Aldrich, SMB00378) were included in culture plates [67]. The ovules were then incubated at 32 °C in the dark without agitation, and TSA (Millipore, 647925) were dissolved in 95% DMSO to make a 1 mM stock solution. Fiber development for all cultural ovules was analyzed after the indicated time of incubation by SEM (Hitachi SU3500). Moreover, the cultured ovules with different times were frozen in liquid nitrogen or at − 80 °C for the following experiments.

### Library preparation, RNA-Seq, and data analysis

Total RNA was extracted from the −2 DPA ovules after 6 days of in vitro culture with TSA and the mock. RNA quantification, qualification, and RNA concentration was measured using NanoDrop 2000 (Thermo). RNA integrity was assessed with the RNA Nano 6000 Assay Kit of the Agilent Bioanalyzer 2100 system (Agilent Technologies, CA, USA).

A total amount of 1 μg RNA per sample was used as input material for the library construction. Sequencing libraries were generated using NEBNext UltraTM RNA Library Prep Kit for Illumina (NEB, USA) following manufacturer’s recommendations by Beijing Biomarker Technologies Co., Ltd (Beijing, China). Six libraries including three biological repeats for each sample were used for the RNA-Seq. Clean data in Illumina sequencing was obtained by removing the containing adapter, containing ploy-N and low quality reads from raw data. Only reads with a perfect match or one mismatch were further analyzed and annotated based on the reference genome. Hisat2 tools was used to map with reference TM-1 (AD1) genome NAU-NBI (Nanjing Agricultural University-Novogene Bioinformatics Technology) assembly v1.1 and annotated v1.1 (https://www.cottongen.org) [68]. The raw data can be accessible from the following BioProject ID: PRJNA733691 in the NCBI SAR database.

Alternative splicing was analyzed with the non-redundant transcript sequences, which were directly used to run all-vs-all BLAST with high identity settings. BLAST alignments that met all criteria were considered products of the candidate AS events: (1) both sequence lengths exceeded 1000 bp and the alignment contained 2 high-scoring segment pairs (HSPs); (2) the alternative splicing gap exceeded 100 bp and was located ≥ 100 bp from the 3′/5′ end; and (3) a 5-bp overlap was allowed for all alternative transcripts.

Gene function was annotated against the databases NR (NCBI non-redundant protein sequences), Nt (NCBI non-redundant nucleotide sequences), Pfam (Protein family) [69], KOG/COG (Clusters of Orthologous Groups of proteins), Swiss-Prot (A manually annotated and reviewed protein sequence database), KO (KEGG Ortholog database) and GO (Gene Ontology) [70].

GATK2 or Samtools software was used to perform SNP calling. Raw vcf files were filtered with GATK standard filter method and other parameters (cluster WindowSize: 10; MQ0 >  = 4 and (MQ0/(1.0*DP)) > 0.1; QUAL < 10; QUAL < 30.0 or QD < 5.0 or HRun > 5), and only SNPs with distance > 5 were retained. Quantification of gene expression levels was estimated by fragments per kilobase of transcript per million fragments mapped. The formula is shown as follow:

$${\text{FPKM = }}\frac{{{\text{cDNA}}\;{\text{Fragments}}}}{{{\text{Mapped Fragments (Millions)}} \times {\text{Transcript Length (kb)}}}}$$

### Differential expression analysis and KEGG pathway enrichment analysis

Differential expression analysis of two samples (Mock and TSA treatments) was performed using the DEseq [71]. Genes with at least twofold change and an adjusted P-value < 0.01 established by DEseq were assigned as differentially expressed. KEGG (Kyoto Encyclopedia of Genes and Genomes) database and KOBAS software were used to test the statistical enrichment of differential expression genes [72, 73].

### RNA extraction and qRT–PCR

Total RNA from cotton ovules was extracted using the Qiagen RNeasy kit and RNAqueous small-scale phenol-free total RNA isolation kit (Ambion) according to the manufacturer’s instructions and reverse transcribed using the SuperScript RT-PCR system (Invitrogen). The qRT-PCR for each gene was performed in three biological replicates using the KAPA SYBR FAST qPCR Kits (KAPA) and the expression value was quantified and normalized to the value of UBIQUITIN 7 [32]. Mean values and standard errors for each gene were calculated from three biological replicates. Primers are listed in Additional file 4: Table S4.

### Assays of the different phytohormones in ovules after TSA treatments

Ovules of −1 DPA (days post anthesis) were cultured in BT medium supplemented with 10 μM DMSO (mock) or TSA in vitro*.* Samples of 200–500 mg were collected after 5 days of culture and ground into fine powder in liquid nitrogen. Contents of the Plant hormones IAA, TZR, ABA, SA, JA and GA_3_ were determined by high performance liquid chromatography and mass spectrometry (HPLC–MS/MS) (KC SEQHEALTH, Wuhan). The ground sample was mixed with acetonitrile solution (v/v), each containing 20 ng deuterium-labeled internal standard. Extracted at 4 ℃ overnight, then centrifugation at 12,000*g* for 5 min and the supernatant was taken. The precipitate was added to acetonitrile solution again, and combined to obtain the supernatant. A C18 Sep-Pak column was added and violently shaken for 30 s, and then the supernatant was taken. Purified extract was dried with nitrogen gas and redissolved with 100% methanol. The supernatant was transferred into the HPLC vials and subjected to liquid chromatography–mass spectrometry (LC–MS) analysis on a SCIEX-6500QTRAP LC/MS/MS system, equipped with an ESI Turbo Ion-Spray interface.

## Supplementary Information

**Additional file 1: Table S1.** The gene ID list of 4,209 DEGs identified between mock and TSA-treated ovules.

**Additional file 2: Table S2.** The gene ID list of 94 up-and down-regulated DEGs associated with hormone signal transduction after TSA treatment.

**Additional file 3: Table S3.** The FPKM values of each gene in classifications a, b, c, and d.

**Additional file 4: Table S4. **The Q-PCR primers used in this study.

**Additional file 5:** **Fig. S1.** Application of TSA repress fiber elongation after long-term in vitro culture. **Fig. S2.** MA plot analysis of DEGs in the ovules. **Fig. S3.** Phenotypic observation on initiation and elongation of cultured fibrocytes in vitro under different hormones. **Fig. S4.** Phenotype of cultured fibrocytes in vitro treated with diallyl disulfide.

## Data Availability

Not applicable.

## Abbreviations

ABA: Abscisic Acid
AE: Alternative exon ends
ARF: Auxin response factor
AS: Alternative splicing
BR: Brassinosteroids
CK: Cytokinin
DEGs: Differentially expressed genes
DPA: Days post-anthesis
GA: Gibberellic Acid
GO: Gene Ontology
GSH: Glutathione
HATs: Histone acetyltransferases
HDACs: Histone deacetylases
HDACi: HDAC inhib HDAC inhibitors
IAA: Indole-3-acetic acid inducible
IR: Intron retention
JA: Jasmonic Acid
JAZ: Jasmonate-zim domain
KEGG: Kyoto Encyclopedia of Genes and Genomes
KO: KEGG Ortholog database
KOG/COG: Clusters of Orthologous Groups of proteins
MeJA: Methyl Jasmonate
NcRNAs: Non-coding RNAs
NR: NCBI non-redundant protein sequences
Nt: NCBI non-redundant nucleotide sequences
Pfam: Protein family
SA: Salicylic acid
SAHA: Suberoylanilide hydroxamic acid
SEM: Scanning electron microscopy
Swiss-Prot: A manually annotated and reviewed protein sequence database
TSA: Trichostatin A
TSS: Transcription start site
