# Supplementary material for: Transcriptomic analysis reveals the key role of histone deacetylation via mediating different phytohormone signalings in fiber initiation of cotton
Source: Cell Biosci. 2022 Jul 12;12:107. doi: 10.1186/s13578-022-00840-4 (PMC9277824; doi:10.1186/s13578-022-00840-4)
Supplement: Supplementary file 4 — Additional file 4: Table S4. The Q-PCR primers used in this study. [file 13578_2022_840_MOESM4_ESM.pdf]

Table S4 The Q-PCR primers used in this study

Gene name	Gene ID	QPCR-F	QPCR-R
GhIAA3_A10	Gh_A10G1020	ACCTCTTGCAGGCTCTAGAG AACA	CCTAAGCCCCTGGCTTCTGAA C
GhIAA19_D10	Gh_D10G1512	CCGGACCTCTTGCAGGCTTT AG	CCTAAGCCCCTGGCTTCTGAA C
GhSAUR1_D02	Gh_D02G2216	GCCATGTTGCGGTTTATGTC GG	TGAACCCGAATTCTTCCTCGGC
GhSAUR12_D12	Gh_D12G0291	ACGGCGAAGATGGACTCCCT	TCACAAGGGATGGTAAGGCCC A
GhAUX1_A01	Gh_A01G1955	CTTCCGTGACGCCGCCGTTA	AATCACCACCGGCAGCCGTG
GhSAUR31_A12	Gh_A12G2237	GCAACAACGGTTCGTGATTC CGG	ACGAAACTCCTCGACATGGCA AGG
GhSAUR50_A03	Gh_A03G1766	ACAACACGGCGGTGGCTAC G	TCGGCTCGTCGGAGTAAGCA
GhSAUR8_D02	Gh_D02G2199	ACCCCTCCCTTAGACGTACC	AACTCTTCTTCGGCTCGTCG
GhJAZ1_A08	Gh_A08G2199	AGCTCCGAAAACCCGAAAA CGA	GGTAGATCGCCGGGAATGGAA C
GhJAZ1_D05	Gh_D05G0352	AACCGGCTAGGTCACTGGA GAA	AACAGATTCATGGTCGGAGGC G
GhJAZ10_D02	Gh_D02G1776	TTTGAAGAACCCGCAGGGTC AG	CTGGCAGCAGGGGTAAGAAGA G
GhJAZ10_A03	Gh_A03G1341	TCGATCGCCGTCTAAGCTTT CG	GACCCTGCGGGTTCTTCAAAC T
GhGID1_A08	Gh_A08G1649	CATTCCTCCGCCAATAGTGC CA	GCACAAGGGTACCTATGCTCC G
GhPIF3_A07	Gh_A07G1202	ATGATGGTCCCTTTGCCTTC GG	AGCTGGGGCTCCTGTCATTAG T
GhERF1_D02	Gh_D02G0430	TGACATCGAAAGCCCGACG AAA	TTATCTCCGCCGCGAACTTTCC
GhERF1B	Gh_Sca115107 G01	ATGGGGAAAGTTCGCGGCG G	CCACCACTGGCGAACCTCCG
GhSRK2E_D11	Gh_D11G3472	TGTATGGTCATGCGGGGTTA CG	AAATCAGATGCCGACACTCGG G
GhAHG1_A12	Gh_A12G2380	TGCGGTGGCGACAAACACG T	ATTGCCTTCCCGCCGCGATA
GhPYL5_D10	Gh_D10G2388	CTTAGCTTCAGCGTGGTCGG AG	ACAACGTACGACTCAACGGCA A
GhPYR6_A06	Gh_A06G1418	CCCCTACCACTCTCACGTCC TT	ACAGCGGAGCAACACTGGTTA G
