# Supplementary material for: Transcriptomic analysis reveals the key role of histone deacetylation via mediating different phytohormone signalings in fiber initiation of cotton
Source: Cell Biosci. 2022 Jul 12;12:107. doi: 10.1186/s13578-022-00840-4 (PMC9277824; doi:10.1186/s13578-022-00840-4)
Supplement: Supplementary file 5 — Additional file 5: Fig. S1. Application of TSA repress fiber elongation after long-term in vitro culture. Fig. S2. MA plot analysis of DEGs in the ovules. Fig. S3. Phenotypic observation on initiation and elongation of cultured fibrocytes in vitro under different hormones. Fig. S4. Phenotype of cultured fibrocytes in vitro treated with diallyl disulfide. [file 13578_2022_840_MOESM5_ESM.pdf]

Fig. S1 Application of TSA repress fiber elongation after long-term *in vitro* culture

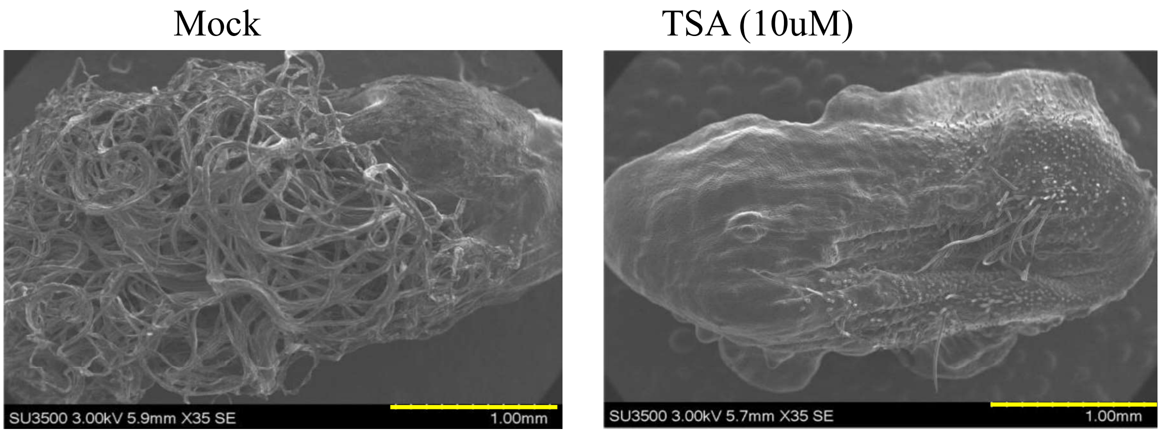

Ovules of 0 DPA were treated with 10 μM TSA for 7 days *in vitro*, and the ovule surface were observed and capture by SEM. Bar= 1 mm (intact ovules).

Fig. S2 MA plot analysis of DEGs in the ovules

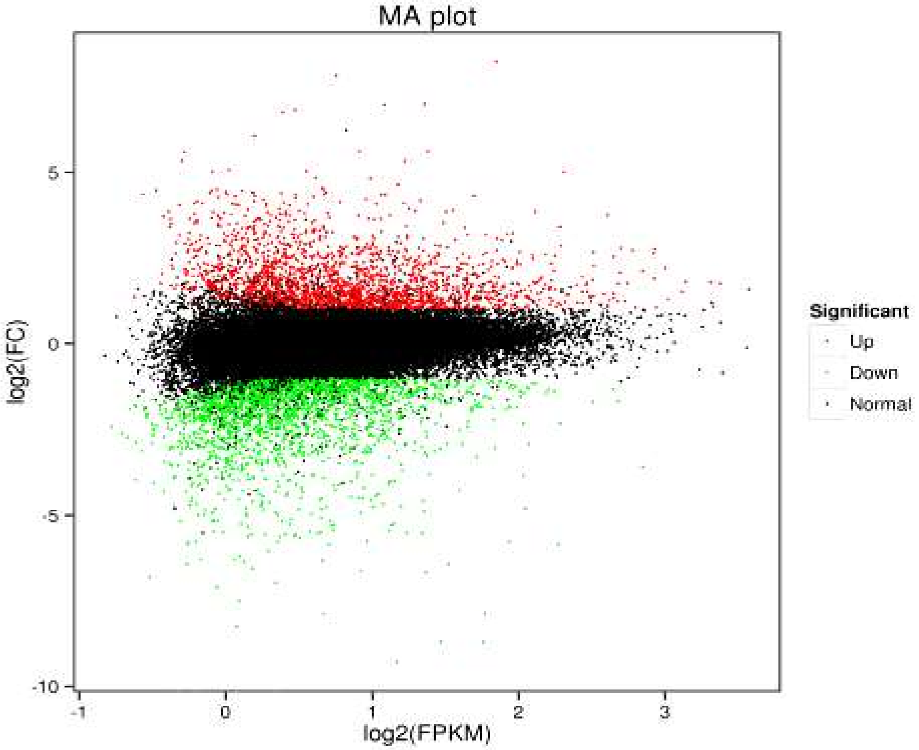

Each dot in the MA map represents one differentially expressed gene. The abscissa is the A value :log2(FPKM) in the two samples,the logarithm value of the mean expression quantity; The ordinate is M value :log2(Fc), which is the logarithm of the fold of gene expression difference between two samples, to measure the expression difference. Green dots represent down-regulated differentially expressed genes, and red dots represent up-regulated differentially expressed genes. The black dots represent non-differentially expressed genes.

Fig. S3 Phenotypic observation on initiation and elongation of cultured fibrocytes in vitro under different hormones

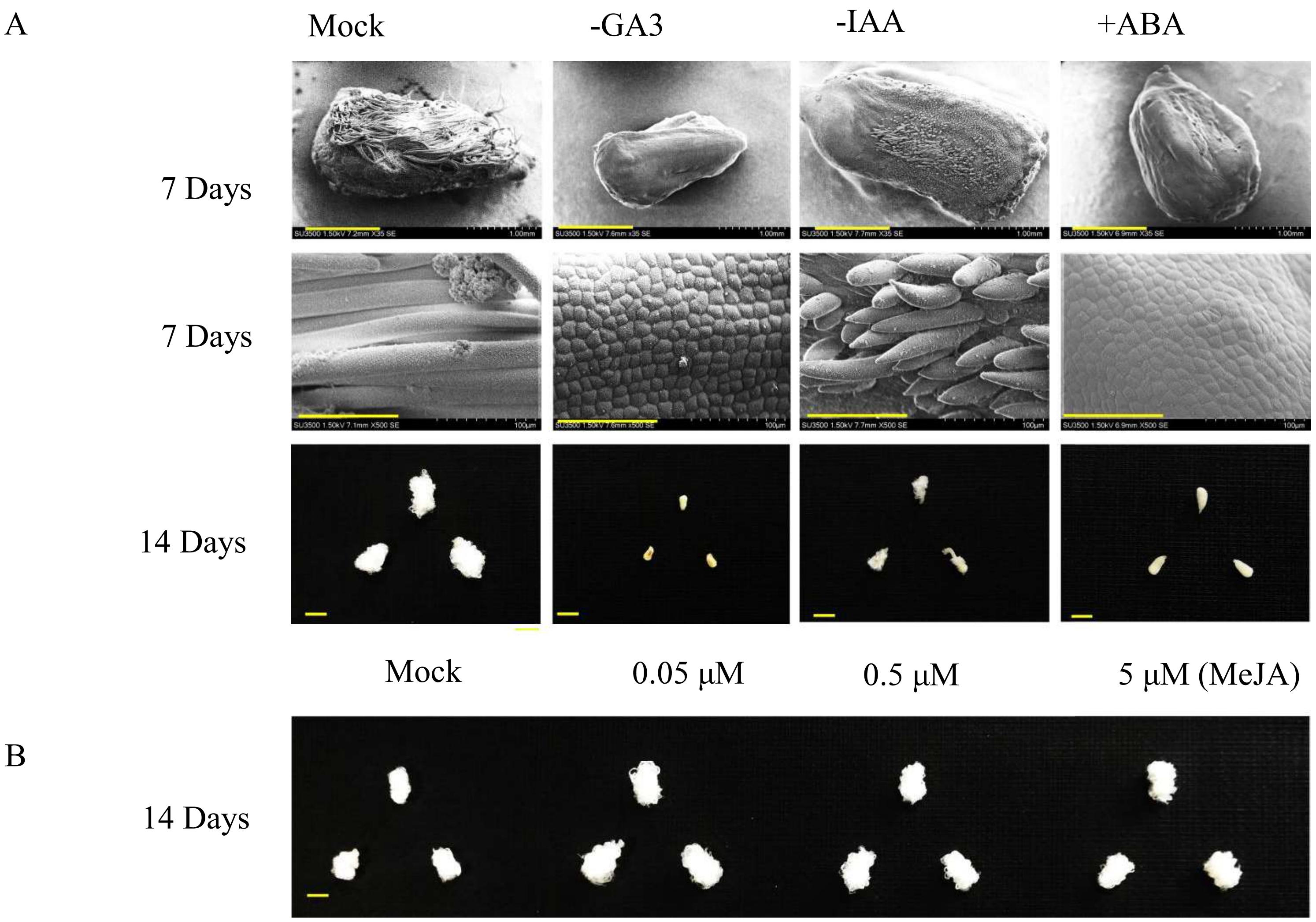

(A) -2 DPA (days post anthesis) ovules were cultured in BT medium without GA3 or IAA and supplemented with 20 μM ABA for 7 days (top and middle) or 14 days (bottom) in vitro, then the ovule surface were observed and captured by Scanning Electron Microscope for 7 days. The pictures on the middle were the magnification of the regions in the top pictures (for ovules cultured at 7 days, Bar=1 mm (intact ovules). Bar=200 μm (magnification), while for 14-day, Bar=2 mm). (B) Effects of different Methyl jasmonate (MeJA)concentrations (0.05, 0.5 μM) on the development of cultured fibers and ovules of ZM24

Fig. S4 Phenotype of cultured fibrocytes *in vitro* treated with diallyl disulfide

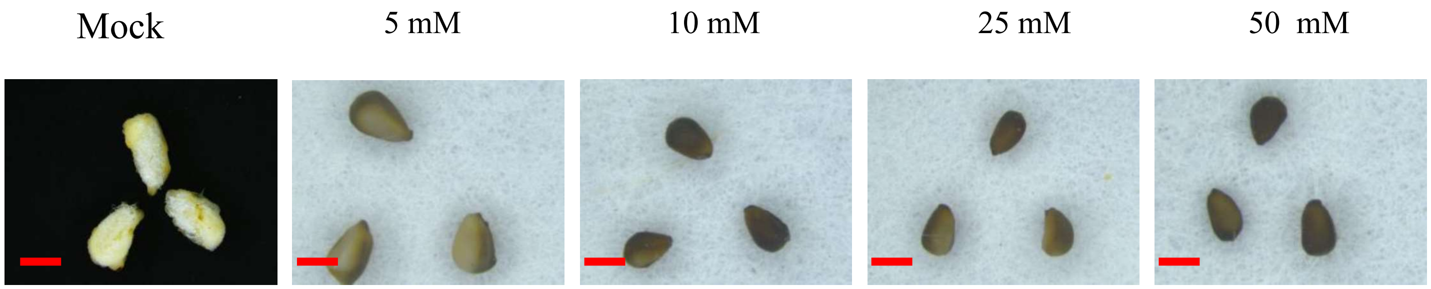

-1 DPA (days post anthesis) ovules were cultured in BT medium containing different concentration gradients of diallyl disulfide (DADC, 0,5, 10,25,50 mM) for 5 days in vitro. Bar=1mm
